# Developing a taxonomy for colorectal cancer

**DOI:** 10.7554/eLife.84025

**Published:** 2022-11-11

**Authors:** Jiansong Fang, Dawei Wang, Xiude Fan

**Affiliations:** 1 Guangzhou University of Chinese Medicine Guangzhou China; 2 Department of Endocrinology, Shandong Provincial Hospital, Shandong University Jinan China; 3 Department of Endocrinology, Shandong Provincial Hospital, Shandong Provincial Hospital Jinan China

**Keywords:** biological network, gene interaction, colorectal cancer, molecular subtype, precision medicine, Human

## Abstract

Analyzing how gene interaction networks are perturbed in individuals can help identify different types of colorectal cancers, paving the way towards personalized care.

**Related research article** Liu Z, Weng S, Dang Q, Xu H, Ren Y, Guo C, Xing Z, Sun Z, Han X. 2022. Gene interaction perturbation network deciphers a high-resolution taxonomy in colorectal cancer. *eLife* **11**:e81114. doi: 10.7554/eLife.81114.

Colorectal cancer is challenging to treat because different patients have widely varying symptoms and the cancers display a broad range of molecular characteristics (Guinney et al., 2015). What can researchers and clinicians do to overcome these obstacles? First, it is necessary to understand the biological properties that make each type of colorectal cancer different, and to establish a precise molecular classification for the different subtypes based on these differences. This will help researchers to develop effective drug interventions. In the past decade, many diseases have been classified based on their transcriptome. However, this ignores the fact that levels of gene expression can vary widely in living patients (Wang et al., 2019).

Now, in eLife, Zhenqiang Sun, Xinwei Han and colleagues at the First Affiliated Hospital of Zhengzhou University – including with Zaoqu Liu as first author – report how they have developed a taxonomy for colorectal cancers based on the interactions between genes rather than on the levels of gene expression (Liu et al., 2022). This approach analyzes networks of gene interactions, which tend to be conserved in healthy samples, but are perturbed in cancer. This makes it possible to characterize the different types of tumors based on how their gene interaction networks are perturbed.

First, the researchers. used a dataset including tumors and healthy samples to generate a large-scale network that had genes as the nodes of the network, and the interactions between genes as the edges. Next, for both healthy and cancerous samples, they quantified the level of perturbation by measuring how the interactions between each pair of genes in the network had changed.

This analysis showed that the perturbations were much stronger in the tumors. Moreover, using a computational approach called clustering analysis, Liu et al. were able to establish six subtypes of colorectal cancers, which they called gene interaction-perturbation network subtypes (or GINS for short; Figure 1). Comparing this new taxonomy with previously published classifications of colorectal cancers revealed that there are notable differences between GINS and subtypes from other taxonomies (Figure 1). Liu et al. were able to confirm the stability and reproducibility of their proposed classification by validating it in independent datasets, which suggests that this taxonomy will be extremely useful for clinical applications.

**Figure 1.**
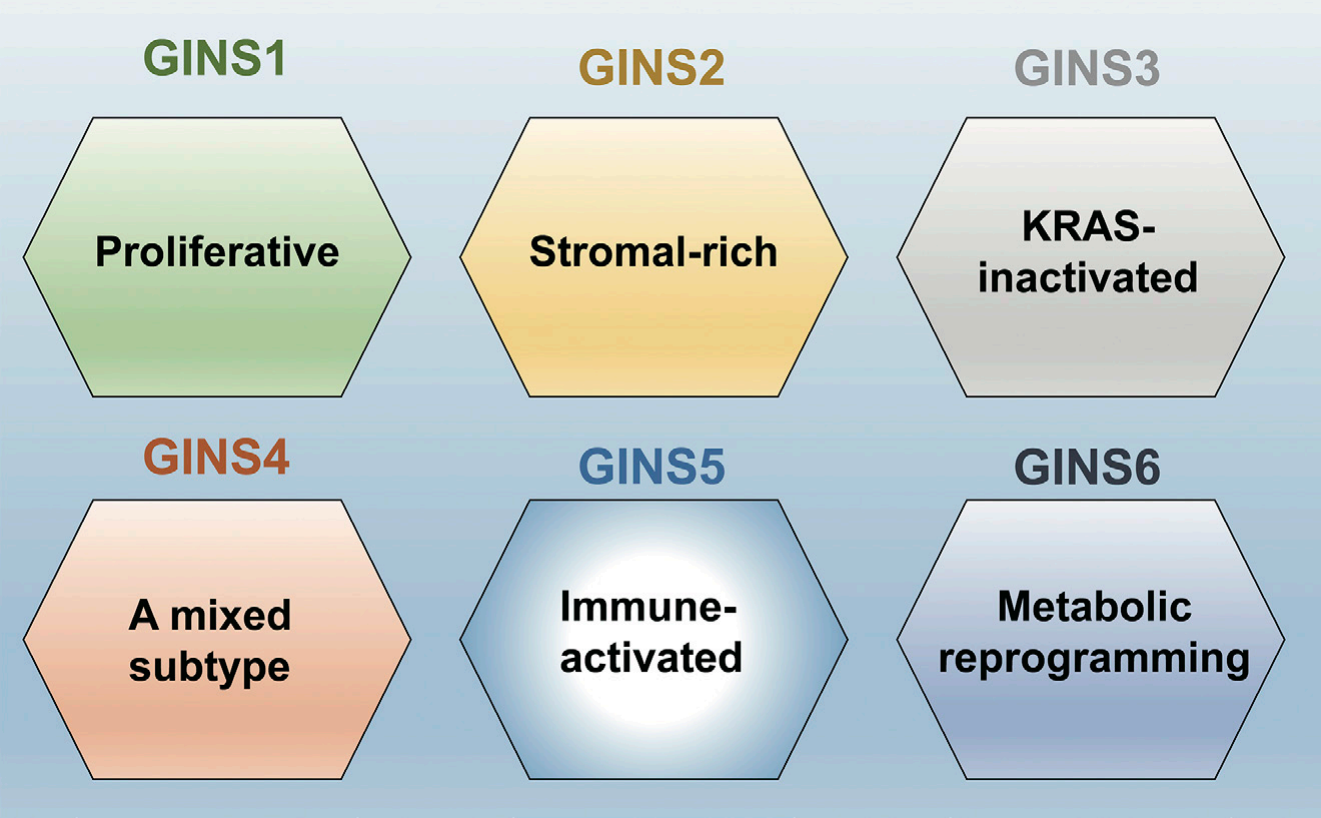
A new taxonomy for colorectal cancer. Liu et al. identified six subtypes of colorectal cancer: GINS1 is a proliferative subtype (green); GINS2 is a stromal-rich subtype (yellow); GINS3 is a KRAS-inactivated subtype (grey); GINS4 is a mixed subtype (red); GINS5 is an immune-activated subtype (bright blue); and GINS6 is a metabolic reprogramming subtype (dark blue). Liu et al. report that GINS4 is an intermediate state between the GINS2 and GINS5 subtypes, which suggests that converting GINS4 into GINS5, which has a better prognosis and may be more sensitive to immunotherapy (especially to PARP inhibitors), might be a good treatment strategy. The GINS6 subtype, on the other hand, may respond well to statins based on its characteristics.

A good molecular classification needs to convey clear biological interpretations of the underlying data, as this will help lay the ground for trials and treatments aimed at specific subtypes (Lin et al., 2021; Ten Hoorn et al., 2022; Singh et al., 2021). Along these lines, Liu et al. report distinguishing features for each of the six subtypes they have identified. For example, GINS1 is a proliferative subtype, whereas GINS2 is a stromal-rich subtype; Figure 1 has details on the other four subtypes.

Looking forward, the new taxonomy will need to be validated by further studies in different populations. Additionally, the classification could be improved by integrating multi-omics data – such as transcriptomic data and proteomic data – into the network. In recent years, the HuRI and BioPlex initiatives have provided comprehensive human interactomes at the proteome level, improving our understanding of genotype-phenotype relationships (Luck et al., 2020; Huttlin et al., 2021). These could be used to make the new perturbation network more robust. Finally, it might also be possible to apply the new network to other challenges, for example, to identify new therapeutic agents for colorectal cancer through network-based approaches (Fang et al., 2021).

The work of Liu et al. is another example of the use of network-based approaches to classify cancer subtypes, such as the recent molecular taxonomy developed for meningioma (Nassiri et al., 2021). The results present and validate a new classification system for colorectal cancer, providing more insights into the heterogeneity of the disease, and facilitating both translational research and personalized approaches to treatment.
